# Sex-Specific Involvement of Estrogen Receptors in Behavioral Responses to Stress and Psychomotor Activation

**DOI:** 10.3389/fpsyt.2019.00081

**Published:** 2019-02-26

**Authors:** Polymnia Georgiou, Panos Zanos, Carleigh E. Jenne, Todd D. Gould

**Affiliations:** ^1^Department of Psychiatry, School of Medicine, University of Maryland, Baltimore, MD, United States; ^2^Department of Pharmacology, School of Medicine, University of Maryland, Baltimore, MD, United States; ^3^Department of Anatomy & Neurobiology, School of Medicine, University of Maryland, Baltimore, MD, United States; ^4^Veterans Affairs Maryland Health Care System, Baltimore, MD, United States

**Keywords:** estrogen receptor alpha, estrogen receptor beta, depressive-like behaviors, mood disorders, sex differences, stress, amphetamine, psychomotor activation

## Abstract

Fluctuating hormone levels, such as estradiol might underlie the difference in the prevalence of psychiatric disorders observed in women vs. men. Estradiol exert its effects primarily through binding on the two classical estrogen receptor subtypes, alpha (ERα) and beta (ERβ). Both receptors have been suggested to a have role in the development of psychiatric disorders, however, most of the current literature is limited to their role in females. We investigated the role of estrogen receptors on cognition (novel-object recognition), anxiety (open-field test, elevated-plus maze, and light/dark box), stress-responsive behaviors (forced-swim test, learned helplessness following inescapable shock, and sucrose preference), pre-pulse inhibition (PPI) and amphetamine-induced hyperlocomotion in both male and female mice either lacking the ERα or ERβ receptor. We found that female *Esr1*^−/−^ mice have attenuated pre-pulse inhibition, whereas female *Esr2*^−/−^ mice manifested enhanced pre-pulse inhibition. No pre-pulse inhibition difference was observed in male *Esr1*^−/−^ and *Esr2*^−/−^ mice. Moreover, amphetamine-induced hyperlocomotion was decreased in male *Esr1*^−/−^, but not *Esr2*^−/−^ mice, while female *Esr1*^−/−^ and *Esr2*^−/−^ mice showed an enhanced response. Genetic absence of ERα did not alter the escape capability or sucrose preference following inescapable shock in both male and female mice. In contrast, female, but not male *Esr2*^−/−^ mice, manifested decreased escape failures compared with controls. Lack of *Esr2* gene in male mice was associated with decreased sucrose preference following inescapable shock, suggesting susceptibility for development of anhedonia following stress. No sucrose preference differences were found in female *Esr2*^−/−^ mice following inescapable shock stress. Lastly, we demonstrated that lack of *Esr1* or *Esr2* genes had no effect on memory and anxiety-like behaviors in both male and female mice. Our findings indicate a differential sex-specific involvement of estrogen receptors in the development of stress-mediated maladaptive behaviors as well as psychomotor activation responses suggesting that these receptors might act as potential treatment targets in a sex-specific manner.

## Introduction

Mental disorders are extremely common, affecting approximately 18.3% of the U.S. adult population (see 2016 National Survey on Drug Use and Health). The prevalence of many mental disorders, including anxiety and depressive disorders, are higher among women than men (see 2016 National Survey on Drug Use and Health). These gender differences have been attributed, at least partly, to fluctuations of the ovarian hormone estradiol [see (1)]. Specifically, an increase in estradiol occurring during female puberty has been associated with increased prevalence of mood disorders [see (2, 3)]. Additionally, several studies showed that the incidence of depression (4–6) and anxiety (7) increases in women during the menopausal transition, a period that is characterized by robust fluctuations in estrogen levels, before overall levels drop to approximately 10% of estrogen levels during the pre-menopausal period. Although estradiol treatment was shown to alleviate depressive symptoms in women (8, 9), the mechanistic relationship between estrogen and depression remains unclear.

Estradiol exerts its effects through binding to two classical estrogen receptor subtypes, the estrogen receptor alpha (ERα) and beta (ERβ) and the non-classical G-protein coupled estrogen receptor, GPR30 (10). These receptors have been suggested to play a role in the pathophysiology of mood disorders. Specifically, *Esr1* gene variants, which code for ERα, have been associated with increased risk of developing depression in women (11–15). In rats, estradiol, via acting at the ERα, normalized postpartum-induced anxiety- and depressive-like behavior, measured in the elevated-plus maze (EPM) and the forced-swim tests (FST), respectively (16). Moreover, knockdown of the ERα selectively in the posterior-dorsal amygdala of female mice decreased anxiety-like behavior as demonstrated by the increase time spend in the light compartment of the light/dark box (L/D box) (17), suggesting a possible role of ERα in regulating anxiety behaviors. Although, the role of ERα has been extensively investigated in women and female animals, its role in male depression and anxiety has received limited attention. However, a genetic association study identified a possible link between *Esr1* polymorphisms and depression in men (18).

Polymorphisms in *Esr2*, which codes for ERβ have been associated with moderate depressive symptoms in women (18), whereas there are no studies investigating the role of *Esr2* polymorphisms in male depression. In rodent studies, selective ERβ ligands (19), as well as estradiol (20) decrease immobility time in the FST in wild-type, but not *Esr2* knockout female mice. In addition, ERβ, but not ERα, agonists decreased immobility time in the FST in ovariectomized rats (21), suggesting that activation of ERβ induces antidepressant effects in female rodents. There is a report suggesting antidepressant efficacy of an ERβ agonist in the tail-suspension test in male mice (22). Administration of the ERβ agonist diarylpropionitrile decreased anxiety-like behaviors in female wild-type mice but not in mice lacking the ERβ receptor gene (23).

Overall, most of the existing studies that have been published and investigated the role of the estrogen receptors in anxiety and depressive behaviors mainly concentrate on a single sex, as estradiol is considered a “female” hormone. Furthermore, limited studies assessed for the involvement of either ERβ or ERα in behavioral responses to stress. Therefore, in the present study, we sought to understand the role of estrogen receptors in anxiety, as well as in depressive-related behavioral responses following stress in both female and male mice.

## Methods

### Mice

*Esr1* and *Esr2* breeding pairs were obtained from Jackson laboratories. Wild-type, heterozygous and homozygous *Esr1* mice were bred in-house by breeding heterozygous males and females. Heterozygous and homozygous *Esr2* mice were bred in-house by breeding heterozygous females and homozygous males. Both *Esr1* and *Esr2* mice were bred on a C57BL/6J background. At the time of behavioral testing, the age of the animals was 8–12 weeks. Mice were grouped-housed and maintained under a 12 h light–dark cycle (lights on at 7:00 a.m.). Water and food was available *ad libitum*. All mice were housed in the same room in individually ventilated cages. All experimental procedures were approved by the University of Maryland Animal Care and Use Committee and were conducted in full accordance with the NIH Guide for the Care and Use of Laboratory Animals. Tail samples were obtained prior to weaning and genotyped by TransnetYX, Inc. (Cordova, TN, USA). The primer sequences are as follows:

*Esr1 genotype: Wild-type*-Forward: TCGGGCATCGCCTACG; Reverse: GGCGACACGCTGTTGAG. *Esr1*-Forward: CATTCTCAGTATTGTTTTGCCAAGTTCT; Reverse: GGCGACACGCTGTTGAG

*Esr2 genotype: Wild-type*-Forward: CCAAGAGGGATGCTCACTTCT: Reverse: CAGACACCGTAATGATACCCAGATG. *Esr2*-Forward: GCCAAGAGGGATGCTCACTTC; Reverse: TCCATCAGAAGCTGACTCTAGAACT.

### Behavioral Characterization

**Figure 1:**
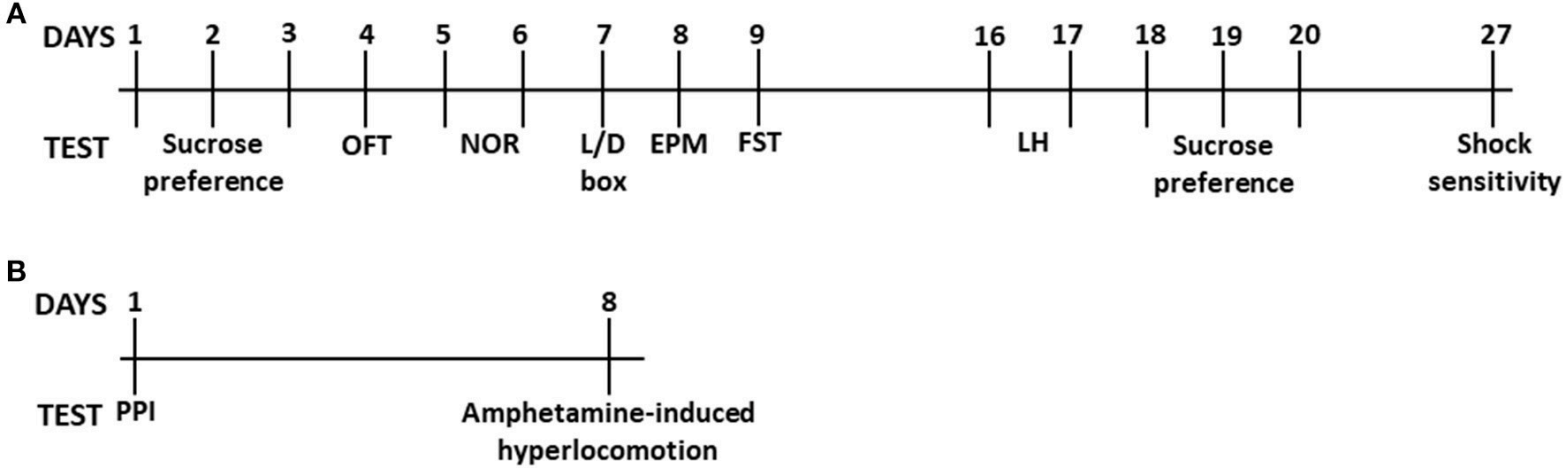
Timeline of behavioral experiments. Timeline of **(A)** the sucrose preference, open-field test, novel-object recognition, light/dark box, elevated-plus maze, forced-swim test, learned helplessness and shock sensitivity. Timeline of **(B)** pre-pulse inhibition and amphetamine-induced hyperlocomotion.

All behavioral experiments were performed during the light phase of the light/dark cycle between 10:00 a.m. and 3:00 p.m. The order of testing within animals (Figure 1A) was determined by the degree of stressfulness of each test with the least stressful conducted first and the most potentially distressing test last (24–26). The numbers of animals used for the open-field test, light/dark box, elevated-plus maze, novel-object recognition, forced-swim test, learned helplessness, shock sensitivity and sucrose preference are as follows—*Esr1*^+/+^, *Esr1*^+/−^, *Esr1*^−/−^ Females: *n* = 12, 20, 11; Males: *n* = 8, 12, 9 and *Esr2*^+/−^, *Esr2*^−/−^ Females: *n* = 9, 8; Males: *n* = 13, 11. The numbers of animals used for the pre-pulse inhibition and amphetamine-induced hyperlocomotion are as follows—*Esr1*^+/+^, *Esr1*^+/−^, *Esr1*^−/−^ Females: *n* = 11, 11, 10; Males: *n* = 10, 10, 8 and *Esr2*^+/−^, *Esr2*^−/−^ Females: *n* = 11, 9; Males: *n* = 7, 8.

#### Open-Field Test (OFT)

The OFT was performed under 300 Lux white lighting. Mice were individually placed into open-field arenas (100 × 100 × 38 cm; San Diego Instruments, San Diego, CA) for a 10-min period. The sessions were recorded using an overhead, digital video-camera. Distanced traveled and time spent in the center of the arena was analyzed using TopScan v2.0 (CleverSys, Inc., Reston VA).

#### Light/Dark Box (L/D box)

The L/D box was used as previously described (26), with minor modifications. Briefly, mice were placed in the illuminated compartment of the L/D box (35 × 35 cm), facing the wall opposite to the dark compartment, and allowed to explore the whole apparatus for 5 min. The sessions were recorded using a video-camera and the time spent in the illuminated and dark compartment was scored using TopScan v2.0 (CleverSys, Inc., Reston VA).

#### Elevated-Plus Maze (EPM)

The EPM was carried in dim white lighting conditions (~5 lux). The apparatus consisted of 2 closed arms and 2 open arms (39 × 5 cm each) and was elevated 50 cm above the floor (Stoelting, Woodale, IL). The experiment was carried out as previously described (27). The time spent in the open and closed arms of the EPM during the 5-min test was recorded by an over-head digital video-camera and scored using TopScan v2.0 (CleverSys, Inc., Reston VA). Amount of time spent in the open arms was used as the primary outcome for the anxiety behavioral assessment.

#### Novel-Object Recognition (NOR)

Short-term recognition memory was assessed using the novel object recognition task protocol, as previously described (28, 29). The NOR was carried in dim white lighting conditions (~10–15 lux). The apparatus and objects used here has been previously described by Zanos et al. (28). The test was conducted over two days. On the first day, the habituation phase, the animals were allowed to explore an empty novel object recognition apparatus (40 × 9 × 23 cm) for 30 min and then returned to their home cages. On the second day, the mice were re-introduced into the same apparatus, but this time containing two identical objects fixed on the floor, which they were allowed to explore for 30 min. After this familiarization phase, mice were immediately returned to their home-cages for 30 min. The mice were then placed back into the novel object recognition apparatus, in which one of the “familiar” objects was replaced by a “novel” object (retention phase) for 4 min. All three phases of the novel object recognition test were recorded via an overhead video-camera and analyzed using TopScan v2.0 automated scoring software (CleverSys, Inc., Reston VA). The time spent interacting with the familiar and novel objects during the retention phase was measured. A discrimination ratio was calculated by dividing the time of interaction with the novel object by the total time of interaction with both objects during the retention phase.

#### Forced-Swim Test (FST)

The FST was performed in normal white light conditions (~300 lux) and was performed as previously described (30). Briefly, mice were subjected to a 6-min swim session in clear Plexiglas cylinders (30-cm height × 20-cm diameter) filled with 15 cm of water (23 ± 1°C). Sessions were recorded using a digital video camera. Immobility time, defined as passive floating with no additional activity other than that necessary to keep the animal's head above water, was scored for the last 4 min of the 6-min test by a trained experimenter blind to the genotypes.

#### Learned Helplessness

The learned helplessness paradigm was performed in accordance with Dao et al. (31) and was separated in two phases: training and test. On Day 1 mice received inescapable shock training (0.3 mA, 2 s shock duration 120 trials, inter-trial interval 15 s) in one compartment of the two-compartment Coulbourn Mouse Shuttle Cage (Coulbourn Instruments, Whitehall, PA). On day 2, learned helplessness test consisted of 45 escapable shock trials (0.3 mA, duration of open door: 15 s). The average inter-trial interval was 20 s. In trials 1–5, the gate opened concomitantly with the shock initiation and stayed open for the duration of the shock. In trials 6–45, the gate with a 3 s delay after initiation of the shock. In all trials, shock was terminated if mice passed through the gate to the other compartment. Number of escape failures and escape latency was automatically measured by GraphicState 3.01 (Coulbourn Instruments, Whitehall, PA).

#### Shock Sensitivity Test

Mice were tested for their sensitivity to shock as previously described with minor modifications (32). Briefly, mice were subjected to increment shock intensities (0.02–0.50 mA; 0.02 mA increments from 0.02 to 0.2 mA and 0.5 mA increments from 0.2 to 0.5 mA) and testing for their flinch response. The increments occurred every 30 s and the shock delivery were automatically controlled by GraphicState 3.01 (Coulbourn Instruments, Whitehall, PA). Scoring was performed live by an experienced experimenter blind to the genotypes.

#### Sucrose Preference Test

For assessing the baseline sucrose preference, mice were singly housed for 72 h and presented with two identical bottles containing either tap water or 1% sucrose solution. After baseline sucrose measurement, mice were re-group housed. Following the learned helplessness testing, similar to the baseline measurements, mice were singly housed for 72 h and presented with two identical bottles containing either tap water or 1% sucrose solution. The location of the sucrose and tap water bottle was changed every day to avoid the development of side preference.

#### Pre-pulse Inhibition (PPI)

The pre-pulse inhibition (PPI) paradigm was performed as previously described (33), with minor modifications. Mice were individually tested in acoustic startle boxes (SR-LAB; San Diego Instruments, San Diego, CA). The animals were placed in the startle chamber for a 30-min habituation period. The experiment started with a further 5-min adaptation period during which the mice were exposed to a constant background noise (67 db), followed by five initial startle stimuli (120 db, 40-ms duration each). Subsequently, animals were exposed to five different trial types: pulse alone trials (120 db, 40-ms duration), three prepulse trials of 76, 81, and 86 db of white noise bursts (20-ms duration) preceding a 120-db pulse by 100 ms, and background (67 db) no-stimuli trials. Each of these trials was randomly presented five times. The percentage PPI was calculated using the following formula: [(magnitude on pulse alone trial—magnitude on prepulse + pulse trial)/magnitude on pulse alone trial] × 100.

#### Amphetamine-Induced Hyperlocomotion

The amphetamine-induced hyperlocomotion experiment was performed under white lighting conditions of ~80 lux. Mice were placed into the open-field arenas (50 × 50 × 38 cm; San Diego Instruments) for a 30-min habituation period, as described in the OFT protocol above. Following the habituation period, the locomotion response to a saline injection (5 ml/kg, i.p.) was assessed for 30-min. After that, mice were administered *d-*amphetamine (2 mg/kg, i.p.; Sigma-Aldrich, St. Louis, MO) and placed back to the arena for 60-min to assess their locomotion response. Distanced traveled and time spent in the center of the arena was analyzed using TopScan v2.0 (CleverSys, Inc., Reston VA).

All the behavioral assessments were performed by an experimenter blind to the genotype of animals. The OFT, novel object recognition, L/D box, EPM, FST, learned helplessness, sucrose preference and shock sensitivity were performed on the same animals starting from the least to the most stressful test (for timeline see Figure 1A). There was a 7-day gap period between the FST and learned helplessness and a 7-day gap between sucrose preference post-stress and shock sensitivity, when mice remained undisturbed in their home cages. The PPI and amphetamine-induced hyperlocomotion was performed on the same animals with at least a 7-day gap between these tests (for timeline see Figure 1B).

### Statistical Analysis

The OFT, L/D box, EPM, novel object recognition, FST, and acoustic startle as well as, the total escape failures in the learned helplessness test with the wild-type, heterozygous and homozygous *Esr1* mice were analyzed using one-way ANOVA. The OFT, L/D box, EPM, novel object recognition, FST, and acoustic startle as well as, the total escape failures in the learned helplessness test with the heterozygous and homozygous *Esr2* mice were analyzed with an unpaired Student's *t*-test. The average escape latency, escape failures, sucrose preference, pre-pulse inhibition and amphetamine-induced hyperlocomotion for both *Esr1* and *Esr2* were analyzed with a repeated measure two-way ANOVA. Datasets that fail to pass normality test as tested by Shapiro-Wilk test, were analyzed by Kruskal-Wallis and Mann-Whitney test. Fisher's exact test was used as an additional analysis for % pre-pulse inhibition to test whether the proportion of mice decreased their PPI was significantly different between genotypes. The male and female mice experiments were performed in separate cohorts, and thus are not combined on the same graphs and statistical analyses. Holm-Sidak *post-hoc* test was performed when a significant interaction effect was observed in the ANOVAs. Statistical significance was set at *p* < 0.05. All analyses were performed using GraphPad Prism *v* 6.01. All values are expressed as mean ± SEM. Statistical details are summarized in Table 1.

**Table 1:** Details of statistical analyses.

**OVERALL EFFECTS FOR FIGURE 2**						
**Males**						
Distance traveled	*F*_(2, 26)_ = 1.703	*P =* 0.202				
Time in center	*F*_(2, 26)_ = 0.308	*P =* 0.738				
Light/Dark Box	*F*_(2, 24)_ = 0.991	*P =* 0.386				
Elevated-plus maze	*F*_(2, 25)_ = 1.697	*P =* 0.204				
Novel-object recognition	*F*_(2, 24)_ = 0.352	*P =* 0.707				
Forced -swim test	*F*_(2, 26)_ = 0.810	*P =* 0.456				
	**Genotype**		**Intensity**		**Interaction**	
Pre-pulse inhibition	*F*_(2, 25)_ = 0.124	*P =* 0.884	*F*_(2, 50)_ = 1.970	*P =* 0.150	*F*_(4, 50)_ = 0.7864	*P =* 0.540
Amphetamine-induced hyperlocomotion	**Genotype**		**Time**		**Interaction**	
Timeline	*F*_(2, 29)_ = 6.408	*P* = 0.005	*F*_(23, 667)_ = 78.3	*P* < 0.001	*F*_(46, 667)_ = 2.866	*P* < 0.001
Total distance traveled	*F*_(2, 29)_ = 6.408	*P* = 0.005	*F*_(2, 58)_ = 210.8	*P* < 0.001	*F*_(4, 58)_ = 5.628	*P* < 0.001
**Females**						
Distance traveled	*F*_(2, 40)_ = 3.047	*P =* 0.059				
Time in center	*F*_(2, 40)_ = 0.385	*P =* 0.683				
Light/Dark Box	*F*_(2, 40)_ = 0.057	*P =* 0.945				
Elevated-plus maze	*H* = 0.696, *Df* = 2	*P =* 0.706				
Novel-object recognition	*F*_(2, 39)_ = 0.201	*P =* 0.819				
Forced -swim test	*H* = 1.253, *Df* = 2	*P =* 0.535				
Pre-pulse inhibition	*H* = 6.646, *Df* = 8	*P =* 0.575				
Amphetamine-induced hyperlocomotion	**Genotype**		**Time**		**Interaction**	
Timeline	*F*_(2, 29)_ = 1.362	*P* = 0.272	*F*_(23, 667)_ = 66.1	*P* < 0.001	*F*_(46, 667)_ = 2.552	*P* < 0.001
Total distance traveled	*F*_(2, 29)_ = 1.362	*P* = 0.272	*F*_(2, 58)_ = 116.8	*P* < 0.001	*F*_(4, 58)_ = 2.259	*P* = 0.074
**OVERALL EFFECTS FOR FIGURE 3**						
**Males**	**Genotype**		**Time**		**Interaction**	
Escape latency	*F*_(2, 26)_ = 0.698	*P* = 0.507	*F*_(8, 208)_ = 1.246	*P* = 0.274	*F*_(16, 208)_ = 0.211	*P* = 0.999
Escape failures	*F*_(2, 26)_ = 0.774	*P* = 0.472	*F*_(8, 208)_ = 3.763	*P* = 0.000	*F*_(16, 208)_ = 0.186	*P* = 0.999
Total escape failures	*F*_(2, 26)_ = 0.774	*P* = 0.472				
Sucrose preference	*H* = 0.213, *Df* = 2	*P* = 0.213				
**Females**						
Escape latency	*H* = 39.41, *Df* = 26	*P* = 0.046				
Escape failures	*H* = 40.06, *Df* = 26	*P* = 0.039				
Total escape failures	*H* = 7.167, *Df* = 2	*P* = 0.028				
	**Genotype**		**Time**		**Interaction**	
Sucrose preference	*F*_(2, 26)_ = 0.869	*P* = 0.431	*F*_(1, 26)_ = 6.488	*P* = 0.017	*F*_(2, 26)_ = 1.357	*P* = 0.275
**OVERALL EFFECTS FOR FIGURE 4**						
**Males**						
Distance traveled	*t*_(22)_ = 0.170	*P* = 0.867				
Time in center	*t*_(22)_ = 0.273	*P* = 0.787				
Light/Dark Box	*t*_(22)_ = 0.960	*P* = 0.347				
Elevated-plus maze	*t*_(22)_ = 0.433	*P* = 0.669				
Novel-object recognition	*t*_(22)_ = 0.145	*P* = 0.886				
Forced -swim test	*t*_(22)_ = 0.510	*P* = 0.615				
	**Genotype**		**Intensity**		**Interaction**	
Pre-pulse inhibition	*F*_(1, 12)_ = 1.329	*P* = 0.271	*F*_(2, 24)_ = 19.58	*P* < 0.001	*F*_(2, 24)_ = 1.083	*P* = 0.355
Amphetamine-induced hyperlocomotion	**Genotype**		**Time**		**Interaction**	
Timeline	*F*_(1, 13)_ = 0.035	*P* = 0.854	*F*_(23, 299)_ = 57.2	*P* < 0.001	*F*_(23, 299)_ = 0.444	*P* = 0.989
Total distance traveled	*F*_(1, 13)_ = 0.035	*P* = 0.854	*F*_(2, 26)_ = 111.7	*P* < 0.001	*F*_(2, 26)_ = 0.0252	*P* = 0.975
**Females**						
Distance traveled	*t*_(15)_ = 0.619	*P* = 0.545				
Time in center	*t*_(15)_ = 1.216	*P* = 0.243				
Light/Dark Box	*t*_(15)_ = 0.536	*P* = 0.580				
Elevated-plus maze	*U* = 27.0	*P* = 0.416				
Novel-object recognition	*t*_(15)_ = 0.077	*P* = 0.940				
Forced -swim test	*t*_(15)_ = 0.131	*P* = 0.898				
	**Genotype**		**Intensity**		**Interaction**	
Pre-pulse inhibition	*F*_(1, 17)_ = 6.373	*P* = 0.022	*F*_(2, 34)_ = 5.197	*P* = 0.011	*F*_(2, 34)_ = 2.892	*P* = 0.069
Amphetamine-induced hyperlocomotion	**Genotype**		**Time**		**Interaction**	
Timeline	*F*_(1, 13)_ = 2.037	*P* = 0.177	*F*_(23, 299)_ = 149.8	*P* < 0.001	*F*_(23, 299)_ = 2.367	*P* < 0.001
Total distance traveled	*F*_(1, 13)_ = 2.037	*P* = 0.177	*F*_(2, 26)_ = 659.3	*P* < 0.001	*F*_(2, 26)_ = 4.787	*P* = 0.017
**OVERALL EFFECTS FOR FIGURE 5**						
**Males**						
Escape latency	*H* = 27.59, *Df* = 17	*P* = 0.050				
Escape failures	*H* = 28.30, *Df* = 17	*P* = 0.042				
Total escape failures	*U* = 51.50	*P* = 0.254				
	**Genotype**		**Time**		**Interaction**	
Sucrose preference	*F*_(1, 22)_ = 6.001	*P* = 0.023	*F*_(1, 22)_ = 5.644	*P* = 0.027	*F*_(1, 22)_ = 17.63	*P* = 0.000
**Females**						
Escape latency	*F*_(1, 14)_ = 3.489	*P* = 0.083	*F*_(8, 112)_ = 1.674	*P* = 0.113	*F*_(8, 112)_ = 2.116	*P* = 0.040
Escape failures	*F*_(1, 14)_ = 0.947	*P* = 0.347	*F*_(8, 112)_ = 3.068	*P* = 0.004	*F*_(8, 112)_ = 1.219	*P* = 0.294
Total escape failures	*t*_(14)_ = 2.274	*P* = 0.039				
Sucrose preference	*H* = 6.863, *Df* = 3	*P* = 0.076				

## Results

### Effects of *Esr1* and *Esr2* Genes on Anxiety-Related Behaviors in Male and Female Mice

#### Open-Field Test

**Figure 2:**
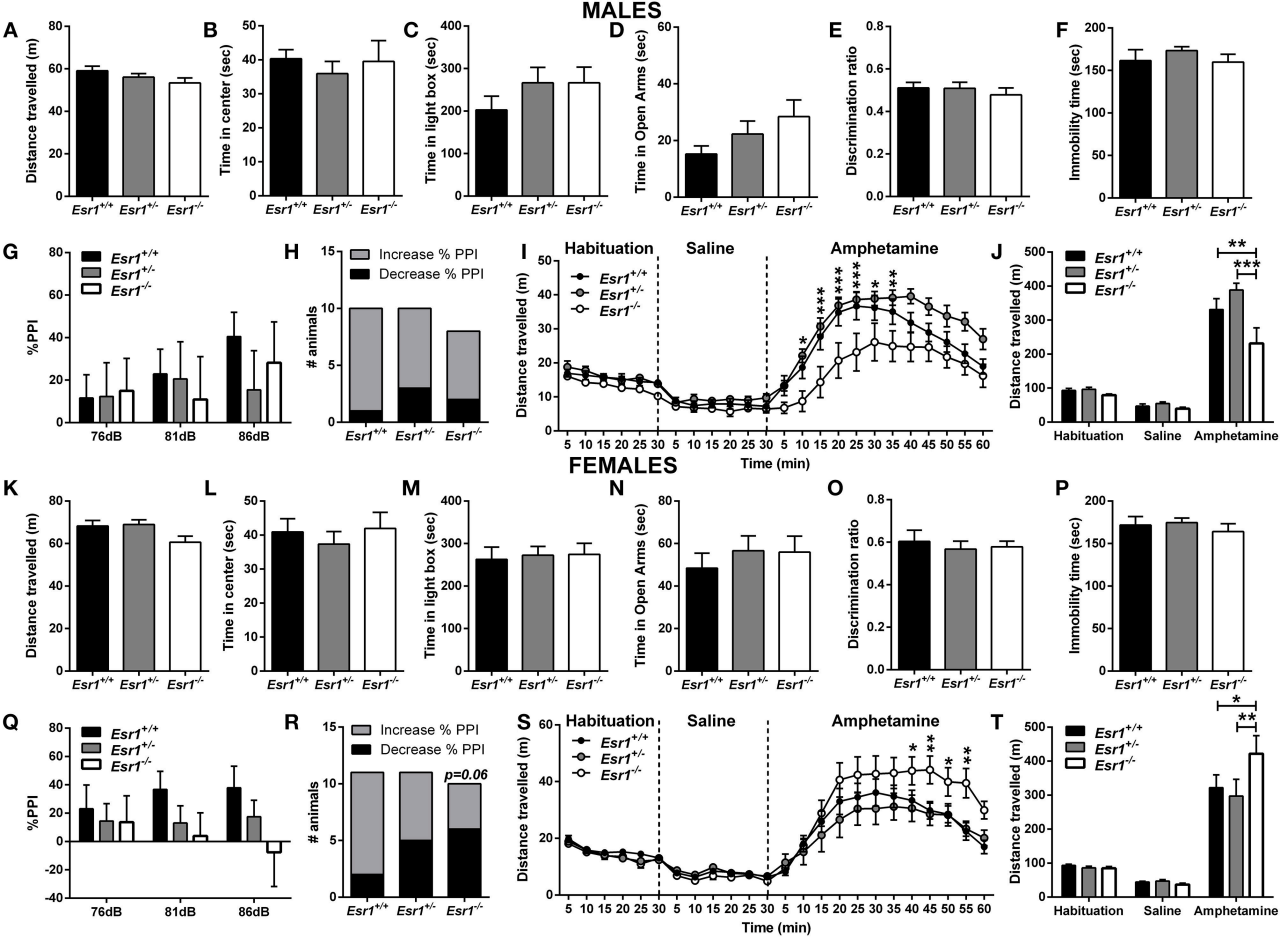
Baseline behavioral characterization of male and female *Esr1*^+/+^, *Esr1*^+/−^ and *Esr1*^−/−^ mice. In male mice, no effect of genotype was observed between in **(A,B)** the open-field test, **(C)** light/dark box, **(D)** elevated-plus maze, **(E)** novel-object recognition, **(F)** forced-swim test, **(G)** pre-pulse inhibition and **(H)** contingency representation of pre-pulse inhibition. **(I,J)** An attenuated amphetamine response was observed in the male *Esr1*^−/−^ mice compared with *Esr1*^+/+^ and *Esr1*^+/−^ mice. In female mice, no genotype difference was observed in **(K,L)** the open-field test, **(M)** light/dark box, **(N)** elevated-plus maze, **(O)** novel-object recognition, **(P)** forced-swim test, **(Q)** pre-pulse inhibition and **(R)** contingency representation of pre-pulse inhibition. **(S,T)** An enhanced amphetamine response was observed in the male *Esr1*^−/−^ mice compared with *Esr1*^+/+^ and *Esr1*^+/−^ mice. ^*^p < 0.05, ^**^p < 0.01, ^***^p < 0.001; *n* = 8, 12, 9 for **(A–F)**, *n* = 10, 10, 8 for **(G–J)**, *n* = 12, 20, 11 for **(K–P)**, *n* = 11, 11, 10 for **(Q–T)**.

We first assessed OFT behavior in male and female heterozygous (*Esr1*^+/−^) and homozygous (*Esr1*^−/−^) *Esr1* mice, as well as their littermate wild-type controls (*Esr1*^+/+^). No difference was observed in the total distance traveled between the groups in both male (Figure 2A) and female mice (Figure 2K). Similarly, no difference was observed in the time-spent in the center of the open field arena in male (Figure 2B) and female mice (Figure 2L).

**Figure 3:**
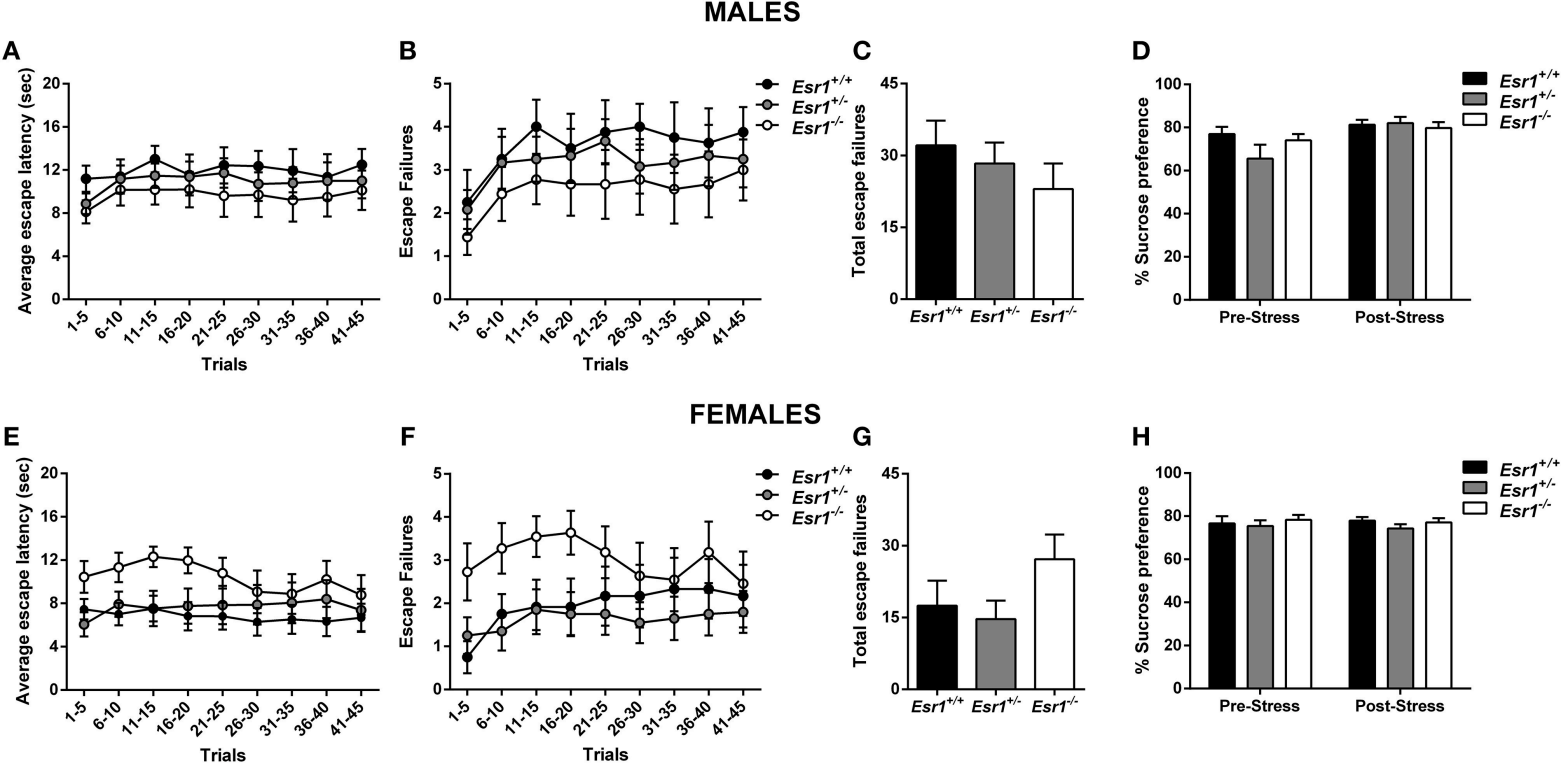
Effects of stress in male and female *Esr1*^+/+^, *Esr1*^+/−^ and *Esr1*^−/−^ mice. In male mice no effect of genotype was observed following inescapable shock training in **(A)** escape latency, **(B,C)** escape failures and **(D)** sucrose preference. In female mice no effect of genotype was observed following inescapable schok training in **(E)** escape latency **(F,G)** escape failures and **(H)** sucrose preference. *n* = 8, 12, 9 for **(A–D)**, *n* = 12, 20, 11 for **(E–H)**.

**Figure 4:**
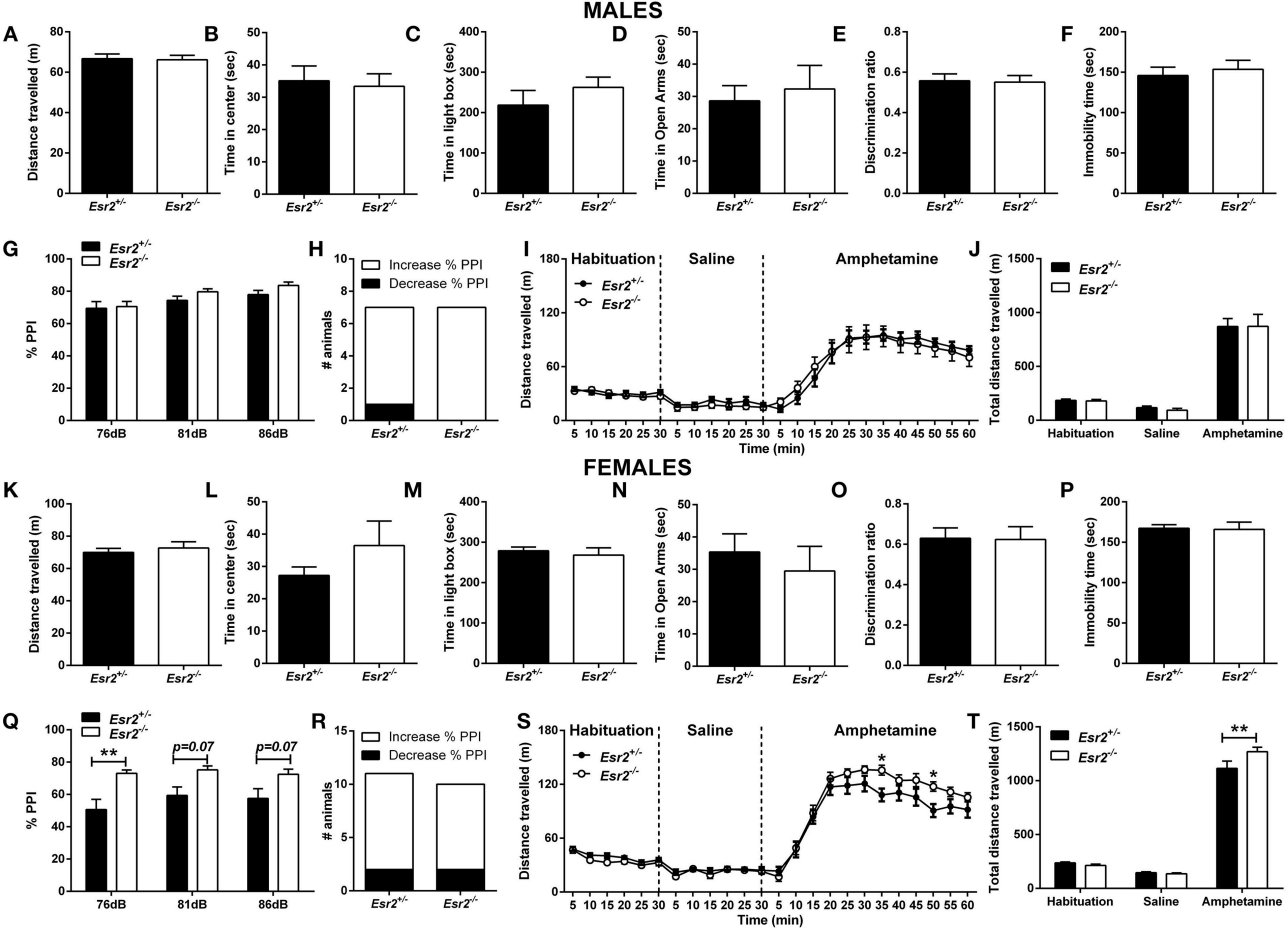
Baseline behavioral characterization of male and female *Esr2*^+/−^ and *Esr2*^−/−^ mice. No difference was observed between the different genotypes in male mice in **(A,B)** the open-field test, **(C)** light/dark box, **(D)** elevated-plus maze, **(E)** novel-object recognition, **(F)** forced-swim test, **(G)** pre-pulse inhibition, **(H)** contingency representation of pre-pulse inhibition and **(I,J)** amphetamine-induced hyperlocomotion. No difference was observed between the different genotypes in female mice in **(K,L)** the open-field test, **(M)** light/dark box, **(N)** elevated-plus maze, **(O)** novel-object recognition and **(P)** forced-swim test. **(Q)** Female *Esr2*^−/−^ mice showed higher pre-pulse inhibition compared with *Esr2*^+/−^ mice. **(R)** No difference was observed in contingency representation of pre-pulse inhibition. **(S,T)** An enhanced amphetamine response was observed in the male *Esr2*^−/−^ mice compared with *Esr2*^+/−^ mice. ^*^p < 0.05, ^**^p < 0.01; *n* = 13, 11 for **(A–F)**, *n* = 7, 8 for **(G–J)**, *n* = 9, 8 for **(K–P)**, *n* = 11, 9 for **(Q–T)**.

Moreover, OFT behavior was assessed in male and female heterozygous (*Esr2*^+/−^) and homozygous (*Esr2*^−/−^) *Esr2* mice. No difference was observed in the total distance traveled between the genotype groups in both male (Figure 4A) and female mice (Figure 4K). Similarly, no difference was observed in the time-spent in the center of the open field arena in male (Figure 4B) and female mice (Figure 4L).

#### Light/Dark Box

We measured the time that mice choose to spend in the brightly illuminated area of the L/D box. No effect of *Esr1* knocked-down (neither *Esr1*^+/−^ or *Esr1*^−/−^) was observed in the in male (Figure 2C) or female (Figure 2M) mice compared with their wild-type littermates.

In addition, both male (Figure 4C) and female (Figure 4M) *Esr2*^−/−^ and *Esr2*^+/−^ mice spent similar time in the illuminated area of the L/D box.

#### Elevated-Plus Maze

We measured the time mice choose to spend in the open arms of the EPM. No difference was observed between the different genotypes (*Esr1*^+/+^, *Esr1*^+/−^, or *Esr1*^−/−^) in male (Figure 2D) and female (Figure 2N) mice.

Also, no difference was observed between the different genotypes in male (Figure 4D) and female (Figure 4N) *Esr2* mice.

### Effects of *Esr1* and *Esr2* Deletion on Novel Object Memory in Male and Female Mice

Short-term recognition memory was assessed using the novel object recognition test. Neither male (Figure 2E) or female (Figure 2O) *Esr1*^−/−^ mice manifest object recognition impairment, since there was no difference in the novel object recognition discrimination ratio compared with the wild-type controls.

Moreover, both male (Figure 4E) and female (Figure 4O) *Esr2* mice did not show any genotype dependent object recognition impairment as assessed with a novel object recognition discrimination ratio.

### Effect of *Esr1* and *Esr2* Deletion on Sensorimotor Gating in Male and Female Mice

Sensorimotor gating deficits were assessed using the PPI paradigm. No statistically significant difference was observed in either male (Figures 2G,H) or female (Figures 2Q,R) *Esr1*^−/−^ mice in the % PPI. However, Fisher's exact test revealed a near significant difference between female *Esr1*^+/+^ and *Esr1*^−/−^ (Figure 2R). No difference was observed between the genotypes in the startle amplitude (Male mice-*Esr1*^+/+^: 250.8 ± 53.14, *Esr1*^+/−^: 235.9 ± 74.03, *Esr1*^−/−^: 270.5 ± 59.86; Female mice-*Esr1*^+/+^: 156.0 ± 30.29, *Esr1*^+/−^: 148.9 ± 32.97, *Esr1*^−/−^: 265.2 ± 63.61).

No difference was observed in male *Esr2*^−/−^ mice in the % PPI (Figures 4G,H) and in the startle amplitude (*Esr2*^+/−^: 231.1 ± 36.26, *Esr2*^−/−^: 205.3 ± 26.94) compared with their littermate controls. However, an increase in the % PPI was observed in female *Esr2*^−/−^ compared with *Esr2*^+/−^ mice (Figure 4Q). No difference was observed in the contingency analysis (Figure 4R) and startle response (*Esr2*^+/−^: 197.3 ± 30.16, *Esr2*^−/−^: 166.6 ± 34.57).

### Effect of *Esr1* and *Esr2* Deletion on *d*-Amphetamine-Induced Hyperlocomotion in Male and Female Mice

An increase in locomotor activity was observed in both male [*F*_(46, 667)_ = 2.866, *p* < 0.001; Figure 2I- *F*_(4, 58)_ = 5.628, *p* < 0.001; Figure 2J] and female mice [*F*_(46, 667)_ = 2.552, *p* < 0.001; Figure 2S- *F*_(4, 58)_ = 2.259, *p* = 0.074; Figure 2T] from all the genotypes. However, male *Esr1*^−/−^ mice showed a lower response to *d*-amphetamine as indicated by the lower distance traveled compared with *Esr1*^+/+^ (*p* < 0.001) and *Esr1*^+/−^ (*p* < 0.01) mice (Figures 2I,J). In contrast, female *Esr1*^−/−^ mice had a greater response to the *d*-amphetamine compared with *Esr1*^+/+^ and *Esr1*^+/−^ mice (Figures 2S,T).

Following administration of *d*-amphetamine, an increase in locomotor activity was observed in both male (Figures 4I,J) and female mice (Figures 4S,T) from all the genotypes. No difference was observed in the *d*-amphetamine-induced hyperlocomotion between male *Esr2*^+/−^ and *Esr2*^−/−^ mice (Figures 4I,J). In contrast, female *Esr2*^−/−^ mice had a greater locomotor response to *d*-amphetamine as shown by the higher distance traveled compared with *Esr2*^+/−^ mice [*F*_(23, 299)_ = 2.367, *p* < 0.001; Figure 4S- *F*_(2, 26)_ = 4.787, *p* = 0.017; Figure 4T].

### Effects of *Esr1* and *Esr2* Deletion on Behavioral Despair and Anhedonia in Male and Female Mice

#### Forced-Swim Test

Behavioral despair was assessed using the FST in male and female *Esr1*^+/−^ and *Esr1*^−/−^ mice, as well as their littermate wild-type controls. Under baseline conditions, no effect of the deletion of *Esr1* was observed in the FST in either male (Figure 2E) or female mice (Figure 2P).

No difference was also observed between *Esr2*^+/−^ and *Esr2*^−/−^ in the immobility time in male (Figure 4F) and female mice (Figure 4P).

#### Learned Helplessness

Development of helpless behavior was tested following inescapable shock. No difference in the escape latency (Figure 3A), and escape failures (Figures 3B,C) was identified between male *Esr1*^+/−^, or *Esr1*^−/−^ mice compared with their littermate wild-type controls. In agreement, there was no difference in escape latency (Figure 3E) and escape failures (Figures 3F,G) between *Esr1*^−/−^, *Esr1*^+/−^ and *Esr1*^+/+^ mice. The learned helplessness response was not affected by differences in shock perception since no differences were observed in the flinch response in the shock sensitivity test by either male (*Esr1*^+/+^: 0.045 ± 0.005, *Esr1*^+/−^: 0.037 ± 0.028, *Esr1*^−/−^: 0.055 ± 0.027) or female (*Esr1*^+/+^: 0.047 ± 0.006, *Esr1*^+/−^: 0.054 ± 0.026, *Esr1*^−/−^: 0.038 ± 0.023) mice.

**Figure 5:**
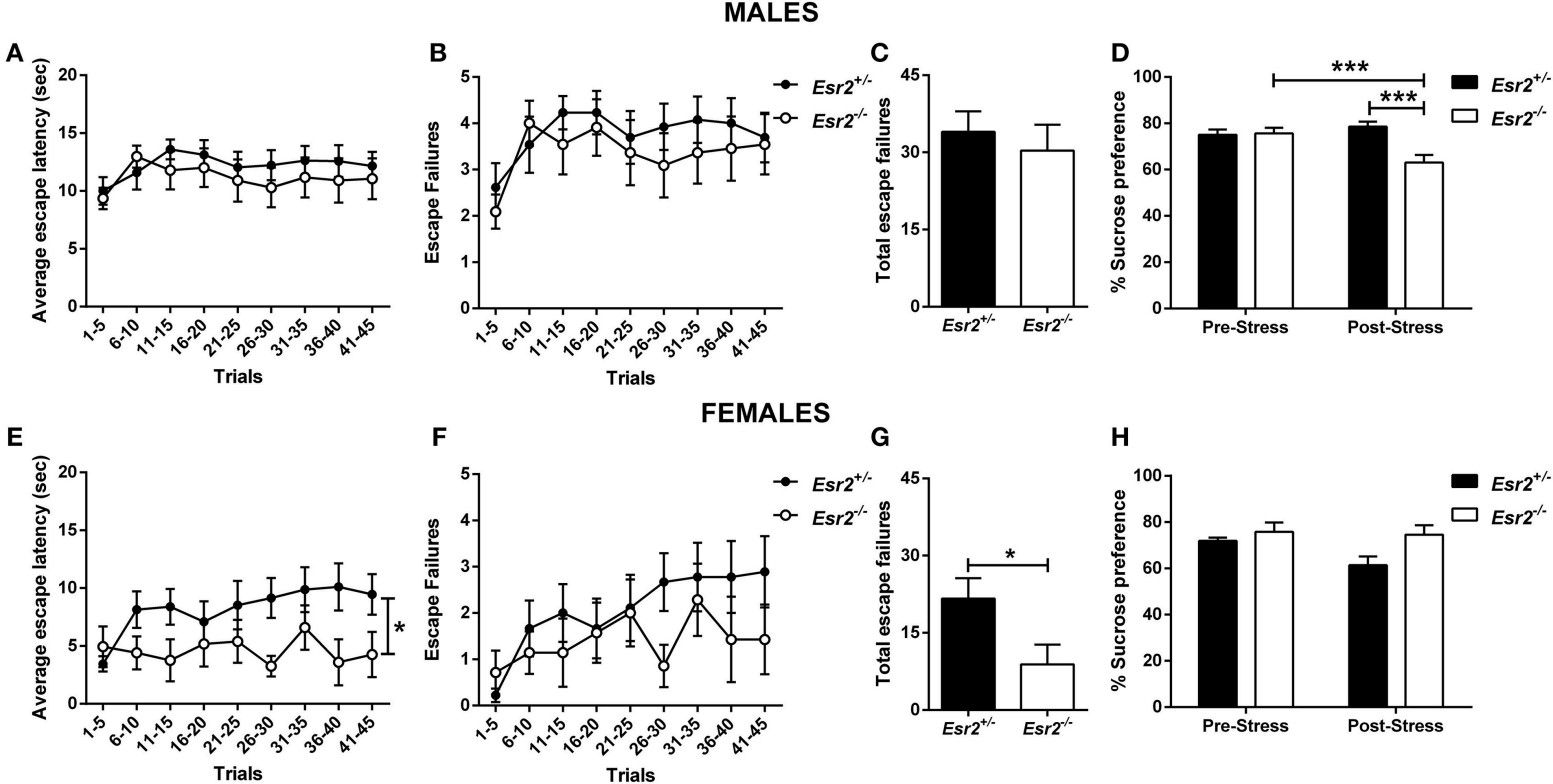
Effects of stress in male and female *Esr2*^+/−^ and *Esr2*^−/−^ mice. No difference was observed between the different genotypes following inescapable shock training in male mice in the **(A)** escape latency and **(B,C)** escape failures. **(D)** Male *Esr2*^−/−^ mice decreased their sucrose preference following stress compared with their baseline and *Esr2*^+/−^ mice. Female *Esr2*^−/−^ mice demonstrated decreased **(E)** escape latency. Although no statistically significant difference was observed in the **(F)** escape failures timeline, **(G)** female *Esr2*^−/−^ mice had lower total escape failures compared with *Esr2*^+/−^ mice. **(H)** No difference was observed in the pre- and post-stress sucrose preference. ^*^p < 0.05, ^***^p < 0.001; *n* = 13, 11 for **(A–D)**, *n* = 9, 8 for **(E–H)**.

No difference in the escape latency (Figure 5A), and escape failures (Figures 5B,C) between the male *Esr2*^+/−^ and *Esr2*^−/−^; while female *Esr2*^−/−^ mice had lower escape latency [*F*_(8, 112)_ = 2.116, *p* = 0.04; Figure 5E] and lower total escape failures [*t*_(14)_ = 2.274, *p* = 0.039; Figure 5G] compared with *Esr2*^+/−^ mice. Although, there was a statistical significance difference in the average escape latency and total escape failures, the breakdown of escape failures does not reach statistical significance (Figure 5F). The learned helplessness response was not affected by any differences in shock perception since no differences were observed in the flinch response in the shock sensitivity test by both male (*Esr2*^+/−^: 0.057 ± 0.007, *Esr2*^−/−^: 0.062 ± 0.007) and female (*Esr2*^+/−^: 0.091 ± 0.005, *Esr2*^−/−^: 0.090 ± 0.007) mice.

#### Sucrose Preference

The development of anhedonia was tested at baseline (stress-naïve) conditions and following footshock stress (as described in the learned helplessness experiment) in male and female *Esr1*^+/−^ and *Esr1*^−/−^ mice, as well as wild-type control mice. No differences were observed between the different genotypes at baseline sucrose preference following stress in either male (Figure 3D) or female mice (Figure 3H).

In addition, the development of anhedonia was tested at baseline and following the learned helplessness procedure in male and female *Esr2*^+/−^ and *Esr2*^−/−^ mice. Although no difference was observed in sucrose preference prior to learned helplessness, following stress, male *Esr2*^−/−^ mice decreased their sucrose preference compared with controls as well as compared with their pre-stress sucrose preference [*F*_(1, 22)_ = 17.63, *p* < 0.001; Figure 5D]. No difference was observed in female *Esr2*^−/−^ and *Esr2*^+/−^ mice was observed both before and after stress (Figure 5H).

## Discussion

In the present study, we demonstrate that the lack of ERα and ERβ are differentially involved in the development of helplessness and anhedonia in male and female mice following stress. To our knowledge, this is the first study to investigate the effects ERα and ERβ using knockout mice in the development of helplessness, i.e., increase in escape failures following inescapable shock training, as well as the development of anhedonia following stress in both male and female mice. We demonstrate that female *Esr2*^−/−^ mice manifested significantly lower escape failures in the learned helplessness test, as well as an overall higher sucrose preference prior and after the learned helplessness stress compared with the heterozygous controls suggesting that deletion of *Esr2* gene might be beneficial against the development of maladaptive behaviors following stress. Our finding is not in line with previous findings that administration of ERβ agonists in ovariectomized female rats decrease immobility time in the FST (21, 34). The different approaches used to investigate the role of ERβ in responses to stress between the aforementioned and present study might account for these differences. For example, here we investigated the development of helplessness following the exposure of mice to stress (inescapable shock), whereas the previously published reports used the FST in stress-naïve rats as a measure of antidepressant efficacy of ERβ agonists. Moreover, our use of intact mice vs. the use of ovariectomized rats in this earlier study might also contribute to these differences.

While we demonstrate that deletion of *Esr2* gene in female mice has a protective effect against the development of helplessness, this is not the case in male *Esr2*^−/−^ mice, which had similar escape failures compared to the *Esr2*^+/−^ mice. However, we observed a decrease in sucrose preference following inescapable shock stress in male *Esr2*^−/−^ compared with *Esr2*^+/−^ control mice, suggesting that *Esr2*^−/−^ male mice are more susceptible to stress-induced anhedonia. To our knowledge this is the first study to demonstrate a role of *Esr2* gene in male mice in the development of stress-induced anhedonia, a core symptom of depression in humans, and further investigation is warranted to identify the specific role of this receptor in male depression.

In contrast with the effects of *Esr2* deletion, following inescapable shock, we did not observe any statistically significant differences in either male or female *Esr1*^−/−^ mice compared with either wild-type and heterozygous littermates. Moreover, neither male nor female *Esr1*^−/−^ mice showed any anhedonia symptoms as measured by sucrose preference prior or after the inescapable shock stress suggesting that *Esr1* is not substantially involved in the development of these behavioral responses. These conclusions are also in agreement with our finding that both male and female *Esr1*^−/−^ mice do not manifest any differences in the FST compared with their wild-type controls. It has been recently reported that ERα in the nucleus accumbens drives a pro-resilient phenotype in both male and female mice (35). This is in apparent contrast with our findings demonstrating that lack of *Esr1*^−/−^ does not induce susceptibility to develop learned helplessness or result in changes in sucrose preference following stress in either sex. It may be that while increased ERa driven transcription mediates resilience, lack of the gene does not modulate susceptibility.

Although we demonstrated differential effects of both estrogen receptors in male and female mice in response to stress, we did not observe any effects of genetic deletion of estrogen receptors in anxiety-related tests. In line with our findings, Krezel et al. (36) demonstrated that both male and female *Esr1*^−/−^ mice had similar thigmotaxis and spent similar time in open arms of the EPM compared with wild-type mice. While, our findings in male *Esr2*^−/−^ mice are also in agreement with other reports (36, 37), their finding that female *Esr2*^−/−^ mice have higher anxiety-like behaviors compared with wild-type mice, as measured by the EPM and OFT, are in contrast with the results presented here. In line with our findings, other reports demonstrated that female *Esr2*^−/−^ have similar performance on anxiety behavioral tests as wild-type mice (23, 38). Interestingly, Walf et al. (38) also demonstrated that female wild-type as well as in *Esr2*^−/−^ mice during proestrous compared with diestrous had higher open field-central entries, which was interpreted as an anxiolytic effect. Therefore, future studies should address this limitation and further investigate the effects of the estrous cycle and estrogen receptors in anxiety-like behaviors.

Moreover, another limitation of the current study is the use of heterozygous mice for *Esr2* instead of wild-type as controls. This might be particularly important when negative results are obtained, such as for the anxiety-related behaviors, since heterozygous mice might have different phenotypes than wild-types. However, it was previously shown that *Esr2*^+/−^ behave in a similar manner as wild-type mice in the elevated plus-mice (39), a test assessing anxiety-like behaviors. Considering this, and the fact that our findings are also in agreement with other studies (20, 36, 37, 40) provides confidence to our results. However, for this reason the negative results comparing *Esr2*^+/−^ to *Esr2*^−/−^ should be interpreted cautiously as a full wild-type control was not included in the experimental design. In addition, since we are using conventional knockout mice, the absence of differences in these behaviors could be due to compensatory mechanisms.

Sex differences have been reported in patients with major depression, with male, but not female, patients demonstrating decreased PPI compared with healthy controls (41, 42). In order to test if estrogen receptors might be implicated in these sex differences, we assessed male and female *Esr1* and *Esr2* knockout mice in the PPI paradigm. Although we observed no significant difference with the male *Esr1*^−/−^ and *Esr2*^−/−^ mice, deletion of estrogen receptors in female mice exerted differential effects. Specifically, contingency analysis demonstrated a near significant decrease in % PPI in *Esr1*^−/−^ compared with *Esr1*^+/+^. This may be related to the finding that a decrease in % PPI is observed in rodents following ovariectomy, an effect that is normalized with estradiol replacement (43, 44); thus, considering our results we postulate that this decrease is attributed to the actions of estradiol through ERα. In contrast with the effects of *Esr1* deletion, female *Esr2*^−/−^ mice manifested higher PPI than their littermate controls suggesting an enhanced sensorimotor gating response. In combination with our findings that deletion of *Esr2* gene in female mice results in decreased escape failures, and the literature demonstrating stress-induced decreases in PPI (45–47), these results suggest that deletion of *Esr2* gene in females might result in stress resilience. Enhanced PPI in this case might be also associated with an improved ability of these mice to deal with information processing. Moreover, considering the literature supporting a protective effect of estradiol in women with schizophrenia [see (48)], our findings cannot preclude that the differential regulation of PPI response in *Esr1* and *Esr2* knockout female mice might have a functional relevance to schizophrenia. However, further investigation is warranted for better understanding this finding and the possible implications of estrogen receptors in stress resilience and/or schizophrenia.

Furthermore, we observed that male *Esr1*^−/−^ mice showed attenuated response to amphetamine, as measured by hyperlocomotion, compared with *Esr1*^+/−^ and *Esr1*^+/+^, suggesting a possible interaction between *Esr1*^−/−^ and the dopaminergic signaling. The possible interaction between *Esr1* and the dopaminergic system is also supported by the findings that male mice lacking *Esr1* have decreased tyrosine hydroxylase (TH) mRNA and protein levels in the midbrain (49), which might also explain our findings that male *Esr1*^−/−^ mice manifest attenuated response to amphetamine-induced hyperlocomotion compared with WT mice. A possible interaction between these two systems is further supported by our findings in female mice; however, in this instance lack of *Esr1* in females enhanced the amphetamine-induced hyperlocomotion compared with controls. A decrease in TH mRNA and protein levels in the midbrain was also observed in female mice lacking *Esr1*^−/−^ (49). These differences in the amphetamine-induced locomotion in *Esr1*^−/−^ mice could be related to this finding, or could be influenced by differences in their hormonal status compared with WT mice. Both male and female *Esr1*^−/−^ mice display highly atrophied reproductive organs and are infertile (50–53) and female *Esr1*^−/−^ mice are anovulatory and acyclic (54, 55). Considering that gonadal hormones can affect dopamine response [see (56–58)], the difference in the hormonal status of these mice could contribute to the observed differences in amphetamine-induced hyperlocomotion. An enhancement of amphetamine-induced hyperlocomotion was also observed in female *Esr2* knockout mice compared with their respective controls, whereas no difference was observed in male *Esr2*^−/−^ mice. Female *Esr2*^−/−^ have been reported to have similar levels of TH immunoreactive cells in the midbrain compared to WT mice (59), though this does not preclude differences in dopamine neuron activity or release. Interestingly, ERα and ERβ agonists have been shown to reverse the amphetamine-induced disruption of PPI (60), which further supports a role of these receptors in modulating response to amphetamine. In addition, estradiol is known to modulate several dopamine-related behaviors such as sexual motivation (61), as well as to increase the rewarding effects of *d-*amphetamine (62, 63); however, the exact role of estrogen receptors needs to be further investigated.

Overall, we demonstrate that deletion of either *Esr1* or *Esr2* differentially affects the development of stress-related responses as well as psychomotor responses in male and female mice. Specifically, deletion of *Esr2* in male mice led to increased susceptibility for the development of stress-related maladaptive behaviors, whereas deletion of *Esr2* in female mice resulted in resilience against the development of such behaviors. Also the amphetamine locomotor response was attenuated in male *Esr1*^−/−^ mice, while female *Esr1*^−/−^ and *Esr2*^−/−^ showed enhanced response. The present findings suggest that differential manipulation of *Esr1* and *Esr2* in males and females might have potential applications for the treatment of mood disorders.

## Author Contributions

PG and TG designed the experiment. PG, PZ, and CJ performed the experiments presented in this manuscript. PG analyzed all the results included in this manuscript. PG and TG drafted the paper. PZ and CJ critically reviewed the manuscript.

### Conflict of Interest Statement

The authors declare that the research was conducted in the absence of any commercial or financial relationships that could be construed as a potential conflict of interest.

## References

[1] Wharton W Gleason CE Olson SR Carlsson CM Asthana S. Neurobiological underpinnings of the estrogen - mood relationship. Curr Psychiatry Rev. (2012) 8:247–56. 10.2174/157340012800792957 23990808 PMC3753111

[2] Altemus M Sarvaiya N Neill Epperson C. Sex differences in anxiety and depression clinical perspectives. Front Neuroendocrinol. (2014) 35:320–30. 10.1016/j.yfrne.2014.05.004 24887405 PMC4890708

[3] Balzer BW Duke SA Hawke CI Steinbeck KS. The effects of estradiol on mood and behavior in human female adolescents: a systematic review. Eur J Pediatr. (2015) 174:289–98. 10.1007/s00431-014-2475-3 25567794

[4] Matthews KA. Myths and realities of the menopause. Psychosom Med. (1992) 54:1–9. 10.1097/00006842-199201000-00001 1553395

[5] Hardy R Kuh D. Change in psychological and vasomotor symptom reporting during the menopause. Soc Sci Med. (2002) 55:1975–88. 10.1016/S0277-9536(01)00326-4 12406465

[6] Woods NF Mariella A Mitchell ES. Patterns of depressed mood across the menopausal transition: approaches to studying patterns in longitudinal data. Acta Obstet Gynecol Scand. (2002) 81:623–32. 10.1034/j.1600-0412.2002.810708.x 12190837

[7] Paoletti AM Floris S Mannias M Orru M Crippa D Orlandi R. Evidence that cyproterone acetate improves psychological symptoms and enhances the activity of the dopaminergic system in postmenopause. J Clin Endocrinol Metab. (2001) 86:608–12. 10.1210/jcem.86.2.7179 11158017

[8] Schmidt PJ Nieman L Danaceau MA Tobin MB Roca CA Murphy JH. Estrogen replacement in perimenopause-related depression: a preliminary report. Am J Obstet Gynecol. (2000) 183:414–20. 10.1067/mob.2000.106004 10942479

[9] Soares CN Almeida OP Joffe H Cohen LS. Efficacy of estradiol for the treatment of depressive disorders in perimenopausal women: a double-blind, randomized, placebo-controlled trial. Arch Gen Psychiatry (2001) 58:529–34. 10.1001/archpsyc.58.6.529 11386980

[10] Yasar P Ayaz G User SD Gupur G Muyan M. Molecular mechanism of estrogen-estrogen receptor signaling. Reprod Med Biol. (2017) 16:4–20. 10.1002/rmb2.12006 29259445 PMC5715874

[11] Mill J Kiss E Baji I Kapornai K Daroczy G Vetro A. Association study of the estrogen receptor alpha gene (ESR1) and childhood-onset mood disorders. Am J Med Genet B Neuropsychiatr Genet. (2008) 147B:1323–6. 10.1002/ajmg.b.30751 18449864

[12] Kim JJ Pae CU Kim MR Min JA Kim KH Lee CU. Association between estrogen receptor gene polymorphisms and depression in post-menopausal women: a preliminary study. Psychiatry Investig. (2010) 7:224–7. 10.4306/pi.2010.7.3.224 20927313 PMC2947812

[13] Ryan J Scali J Carriere I Peres K Rouaud O Scarabin PY. Oestrogen receptor polymorphisms and late-life depression. Br J Psychiatry (2011) 199:126–31. 10.1192/bjp.bp.111.091751 21804148 PMC3623726

[14] Ryan J Scali J Carriere I Peres K Rouaud O Scarabin PY. Estrogen receptor alpha gene variants and major depressive episodes. J Affect Disord. (2012) 136:1222–6. 10.1016/j.jad.2011.10.010 22051074

[15] Pinsonneault JK Sullivan D Sadee W Soares CN Hampson E Steiner M. Association study of the estrogen receptor gene ESR1 with postpartum depression–a pilot study. Arch Womens Ment Health (2013) 16:499–509. 10.1007/s00737-013-0373-8 23917948 PMC3833886

[16] Furuta M Numakawa T Chiba S Ninomiya M Kajiyama Y Adachi N. Estrogen, predominantly via estrogen receptor alpha, attenuates postpartum-induced anxiety- and depression-like behaviors in female rats. Endocrinology (2013) 154:3807–16. 10.1210/en.2012-2136 23913447

[17] Spiteri T Musatov S Ogawa S Ribeiro A Pfaff DW Agmo A. The role of the estrogen receptor alpha in the medial amygdala and ventromedial nucleus of the hypothalamus in social recognition, anxiety and aggression. Behav Brain Res. (2010) 210:211–20. 10.1016/j.bbr.2010.02.033 20184922

[18] Ryan J Ancelin ML. Polymorphisms of estrogen receptors and risk of depression: therapeutic implications. Drugs (2012) 72:1725–38. 10.2165/11635960-000000000-00000 22901010

[19] Clark JA Alves S Gundlah C Rocha B Birzin ET Cai SJ. Selective estrogen receptor-beta (SERM-beta) compounds modulate raphe nuclei tryptophan hydroxylase-1 (TPH-1) mRNA expression and cause antidepressant-like effects in the forced swim test. Neuropharmacology (2012) 63:1051–63. 10.1016/j.neuropharm.2012.07.004 22796107

[20] Rocha BA Fleischer R Schaeffer JM Rohrer SP Hickey GJ. 17 Beta-estradiol-induced antidepressant-like effect in the forced swim test is absent in estrogen receptor-beta knockout (BERKO) mice. Psychopharmacology (2005) 179:637–43. 10.1007/s00213-004-2078-1 15645223

[21] Walf AA Rhodes ME Frye CA. Antidepressant effects of ERbeta-selective estrogen receptor modulators in the forced swim test. Pharmacol Biochem Behav. (2004) 78:523–9. 10.1016/j.pbb.2004.03.023 15251261

[22] Hughes ZA Liu F Platt BJ Dwyer JM Pulicicchio CM Zhang G. WAY-200070, a selective agonist of estrogen receptor beta as a potential novel anxiolytic/antidepressant agent. Neuropharmacology (2008) 54:1136–42. 10.1016/j.neuropharm.2008.03.004 18423777

[23] Walf AA Koonce CJ Frye CA Estradiol or diarylpropionitrile decrease anxiety-like behavior of wildtype, but not estrogen receptor beta knockout, mice. Behav Neurosci. (2008) 122:974–81. 10.1037/a0012749 18823154 PMC2562611

[24] Crawley JN Paylor R. A proposed test battery and constellations of specific behavioral paradigms to investigate the behavioral phenotypes of transgenic and knockout mice. Horm Behav. (1997) 31:197–211. 10.1006/hbeh.1997.1382 9213134

[25] Crawley JN What's Wrong With My Mouse? Behavioral Phenotyping of Transgenic and Knockout Mice. New York, NY: John Wiley & Sons, Inc, (2006).

[26] Georgiou P Zanos P Hourani S Kitchen I Bailey A. Cocaine abstinence induces emotional impairment and brain region-specific upregulation of the oxytocin receptor binding. Eur J Neurosci. (2016) 44:2446–54. 10.1111/ejn.13348 27453431

[27] Georgiou P Zanos P Garcia-Carmona JA Hourani S Kitchen I Laorden ML. Methamphetamine abstinence induces changes in mu-opioid receptor, oxytocin and CRF systems: association with an anxiogenic phenotype. Neuropharmacology (2016) 105:520–32. 10.1016/j.neuropharm.2016.02.012 26896754

[28] Zanos P Bhat S Terrillion CE Smith RJ Tonelli LH Gould TD. Sex-dependent modulation of age-related cognitive decline by the L-type calcium channel gene Cacna1c (Cav 1.2). Eur J Neurosci. (2015) 42:2499–507. 10.1111/ejn.12952 25989111 PMC4615431

[29] Le NT Chang L Kovlyagina I Georgiou P Safren N Braunstein KE. Motor neuron disease, TDP-43 pathology, and memory deficits in mice expressing ALS-FTD-linked UBQLN2 mutations. Proc Natl Acad Sci USA. (2016) 113:E7580–9. 10.1073/pnas.1608432113 27834214 PMC5127348

[30] Can A Dao DT Arad M Terrillion CE Piantadosi SC Gould TD The mouse forced swim test. J Vis Exp. (2012) 59:e3638 10.3791/3638 PMC3353513 22314943

[31] Dao DT Mahon PB Cai X Kovacsics CE Blackwell RA Arad M. Mood disorder susceptibility gene CACNA1C modifies mood-related behaviors in mice and interacts with sex to influence behavior in mice and diagnosis in humans. Biol Psychiatry (2010) 68:801–10. 10.1016/j.biopsych.2010.06.019 20723887 PMC2955812

[32] Kovacsics CE Gould TD. Shock-induced aggression in mice is modified by lithium. Pharmacol Biochem Behav. (2010) 94:380–6. 10.1016/j.pbb.2009.09.020 19800363

[33] Zanos P Piantadosi SC Wu HQ Pribut HJ Dell MJ Can A. The prodrug 4-chlorokynurenine causes ketamine-like antidepressant effects, but not side effects, by NMDA/GlycineB-site inhibition. J Pharmacol Exp Ther. (2015) 355:76–85. 10.1124/jpet.115.225664 26265321 PMC4576668

[34] Weiser MJ Wu TJ Handa RJ. Estrogen receptor-beta agonist diarylpropionitrile: biological activities of R- and S-enantiomers on behavior and hormonal response to stress. Endocrinology (2009) 150:1817–25. 10.1210/en.2008-1355 19074580 PMC2659273

[35] Lorsch ZS Loh Y-HE Purushothaman I Walker DM Parise EM Salery M. Estrogen receptor α drives pro-resilient transcription in mouse models of depression. Nature Commun. (2018) 9:1116. 10.1038/s41467-018-03567-4 29549264 PMC5856766

[36] Krezel W Dupont S Krust A Chambon P Chapman PF. Increased anxiety and synaptic plasticity in estrogen receptor beta -deficient mice. Proc Natl Acad Sci USA. (2001) 98:12278–82. 10.1073/pnas.221451898 11593044 PMC59805

[37] Tsuda MC Yamaguchi N Nakata M Ogawa S. Modification of female and male social behaviors in estrogen receptor beta knockout mice by neonatal maternal separation. Front Neurosci. (2014) 8:274. 10.3389/fnins.2014.00274 25228857 PMC4151037

[38] Walf AA Koonce C Manley K Frye CA Proestrous compared to diestrous wildtype, but not estrogen receptor beta knockout, mice have better performance in the spontaneous alternation and object recognition tasks and reduced anxiety-like behavior in the elevated plus and mirror maze. Behav Brain Res. (2009) 196:254–60. 10.1016/j.bbr.2008.09.016 18926853 PMC2614898

[39] Walf AA Frye CA. A review and update of mechanisms of estrogen in the hippocampus and amygdala for anxiety and depression behavior. Neuropsychopharmacology (2006) 31:1097–111. 10.1038/sj.npp.1301067 16554740 PMC3624621

[40] Oyola MG Portillo W Reyna A Foradori CD Kudwa A Hinds L. Anxiolytic effects and neuroanatomical targets of estrogen receptor-beta (ERbeta) activation by a selective ERbeta agonist in female mice. Endocrinology (2012) 153:837–46. 10.1210/en.2011-1674 22186418 PMC3275390

[41] Matsuo J Ota M Hidese S Hori H Teraishi T Ishida I. Sexually dimorphic deficits of prepulse inhibition in patients with major depressive disorder and their relationship to symptoms: a large single ethnicity study. J Affect Disord. (2017) 211:75–82. 10.1016/j.jad.2017.01.012 28103521

[42] Matsuo J Ota M Hidese S Teraishi T Hori H Ishida I. Sensorimotor gating in depressed and euthymic patients with bipolar disorder: analysis on prepulse inhibition of acoustic startle response stratified by gender and state. Front Psychiatry (2018) 9:123. 10.3389/fpsyt.2018.00123 29720950 PMC5915895

[43] Charitidi K Meltser I Canlon B. Estradiol treatment and hormonal fluctuations during the estrous cycle modulate the expression of estrogen receptors in the auditory system and the prepulse inhibition of acoustic startle response. Endocrinology (2012) 153:4412–21. 10.1210/en.2012-1416 22778224

[44] Sbisa A Van Den Buuse M Gogos A. The effect of estrogenic compounds on psychosis-like behaviour in female rats. PLoS ONE (2018) 13:e0193853. 10.1371/journal.pone.0193853 29579065 PMC5868772

[45] Pijlman FT Herremans AH Van De Kieft J Kruse CG Van Ree JM Behavioural changes after different stress paradigms: prepulse inhibition increased after physical, but not emotional stress. Eur Neuropsychopharmacol. (2003) 13:369–80. 10.1016/S0924-977X(03)00040-3 12957336

[46] Sutherland JE Conti LH. Restraint stress-induced reduction in prepulse inhibition in Brown Norway rats: role of the CRF2 receptor. Neuropharmacology (2011) 60:561–71. 10.1016/j.neuropharm.2010.12.022 21185316 PMC3162341

[47] De La Casa LG Mena A Ruiz-Salas JC. Effect of stress and attention on startle response and prepulse inhibition. Physiol Behav. (2016) 165:179–86. 10.1016/j.physbeh.2016.07.022 27484698

[48] Gogos A Sbisa AM Sun J Gibbons A Udawela M Dean B. A role for estrogen in schizophrenia: clinical and preclinical findings. Int J Endocrinol. (2015) 2015:615356. 10.1155/2015/615356 26491441 PMC4600562

[49] Kuppers E Krust A Chambon P Beyer C. Functional alterations of the nigrostriatal dopamine system in estrogen receptor-alpha knockout (ERKO) mice. Psychoneuroendocrinology (2008) 33:832–8. 10.1016/j.psyneuen.2008.03.007 18472350

[50] Lubahn DB Moyer JS Golding TS Couse JF Korach KS Smithies O Alteration of reproductive function but not prenatal sexual development after insertional disruption of the mouse estrogen receptor gene. Proc Natl Acad Sci USA. (1993) 90:11162–6. 10.1073/pnas.90.23.11162 8248223 PMC47942

[51] Couse JF Korach KS. Estrogen receptor null mice: what have we learned and where will they lead us? Endocr Rev. (1999) 20:358–417. 10.1210/edrv.20.3.0370 10368776

[52] Hewitt SC Korach KS. Estrogen receptors: structure, mechanisms and function. Rev Endocr Metab Disord. (2002) 3:193–200. 10.1023/A:1020068224909 12215714

[53] Couse JF Yates MM Walker VR Korach KS Characterization of the hypothalamic-pituitary-gonadal axis in estrogen receptor (ER) Null mice reveals hypergonadism and endocrine sex reversal in females lacking ERalpha but not ERbeta. Mol Endocrinol. (2003) 17:1039–53. 10.1210/me.2002-0398 12624116

[54] Lindzey J Korach KS. Developmental and physiological effects of estrogen receptor gene disruption in mice. Trends Endocrinol Metab. (1997) 8:137–45. 10.1016/S1043-2760(97)00007-6 18406799

[55] Sanchez-Andrade G Kendrick KM. Roles of alpha- and beta-estrogen receptors in mouse social recognition memory: effects of gender and the estrous cycle. Horm Behav. (2011) 59:114–22. 10.1016/j.yhbeh.2010.10.016 21056567

[56] Kuhn C Johnson M Thomae A Luo B Simon SA Zhou G. The emergence of gonadal hormone influences on dopaminergic function during puberty. Horm Behav. (2010) 58:122–37. 10.1016/j.yhbeh.2009.10.015 19900453 PMC2883625

[57] Barth C Villringer A Sacher J. Sex hormones affect neurotransmitters and shape the adult female brain during hormonal transition periods. Front Neurosci. (2015) 9:37. 10.3389/fnins.2015.00037 25750611 PMC4335177

[58] Karpinski M Mattina GF Steiner M. Effect of gonadal hormones on neurotransmitters implicated in the pathophysiology of obsessive-compulsive disorder: a critical review. Neuroendocrinology (2017) 105:1–16. 10.1159/000453664 27894107

[59] Johnson ML Ho CC Day AE Walker QD Francis R Kuhn CM. Oestrogen receptors enhance dopamine neurone survival in rat midbrain. J Neuroendocrinol. (2010) 22:226–37. 10.1111/j.1365-2826.2010.01964.x 20136693 PMC3019761

[60] Labouesse MA Langhans W Meyer U. Effects of selective estrogen receptor alpha and beta modulators on prepulse inhibition in male mice. Psychopharmacology (2015) 232:2981–94. 10.1007/s00213-015-3935-9 25893642

[61] Yoest KE Cummings JA Becker JB. Estradiol, dopamine and motivation. Cent Nerv Syst Agents Med Chem. (2014) 14:83–9. 10.2174/1871524914666141226103135 25540977 PMC4793919

[62] Justice AJ De Wit H. Acute effects of d-amphetamine during the follicular and luteal phases of the menstrual cycle in women. Psychopharmacology (1999) 145:67–75. 10.1007/s002130051033 10445374

[63] Justice AJ De Wit H. Acute effects of estradiol pretreatment on the response to d-amphetamine in women. Neuroendocrinology (2000) 71:51–9. 10.1159/000054520 10644899

